# Simultaneous identification of viruses and viral variants with programmable DNA nanobait

**DOI:** 10.1038/s41565-022-01287-x

**Published:** 2023-01-16

**Authors:** Filip Bošković, Jinbo Zhu, Ran Tivony, Alexander Ohmann, Kaikai Chen, Mohammed F. Alawami, Milan Đorđević, Niklas Ermann, Joana Pereira-Dias, Michael Fairhead, Mark Howarth, Stephen Baker, Ulrich F. Keyser

**Affiliations:** 1 Cavendish Laboratory, University of Cambridge, Cambridge, UK; 2 University of Cambridge School of Clinical Medicine, Cambridge Biomedical Campus, Hills Road, Cambridge, UK; 3 Department of Medicine, University of Cambridge School of Clinical Medicine, Cambridge Biomedical Campus, Hills Road, Cambridge, UK; 4 Department of Biochemistry, University of Oxford, Oxford, UK

**Keywords:** Nanopores, DNA nanostructures

## Abstract

Respiratory infections are the major cause of death from infectious disease worldwide. Multiplexed diagnostic approaches are essential as many respiratory viruses have indistinguishable symptoms. We created self-assembled DNA nanobait that can simultaneously identify multiple short RNA targets. The nanobait approach relies on specific target selection via toehold-mediated strand displacement and rapid readout via nanopore sensing. Here we show that this platform can concurrently identify several common respiratory viruses, detecting a panel of short targets of viral nucleic acids from multiple viruses. Our nanobait can be easily reprogrammed to discriminate viral variants with single-nucleotide resolution, as we demonstrated for several key SARS-CoV-2 variants. Last, we show that the nanobait discriminates between samples extracted from oropharyngeal swabs from negative- and positive-SARS-CoV-2 patients without preamplification. Our system allows for the multiplexed identification of native RNA molecules, providing a new scalable approach for the diagnostics of multiple respiratory viruses in a single assay.

## Abstract (web summary)

Polymerase-chain-reaction-based methods used in clinic to identify respiratory viral infections cannot simultaneously detect multiple viruses or viral variants. Here nanopore nucleic acid sensing is coupled to programmable ribonuclease to simultaneously identify specific short RNA sequences of multiple viruses, without predetection steps such as sample purification or preamplification.

## Main

The diagnosis of infectious diseases plays a vital role in determining appropriate patient treatment^1^. Respiratory tract infections are the major cause of death from infectious diseases globally^2,3^. Many respiratory viruses induce comparable symptoms and cannot be clinically differentiated, making the identification of appropriate treatment challenging. It is estimated that 65% of infection-associated cases of pneumonia are potentially misdiagnosed, with 95% of these cases erroneously receiving antimicrobials^4^. The ongoing coronavirus disease 2019 (COVID-19) pandemic further highlights another unmet diagnostic need: the routine identification and screening of viral variants as they arise^5^.

Currently, viral diagnostics rely on quantitative reverse transcription–polymerase chain reaction (qRT-PCR), followed by genome sequencing, to detect viral variants^5,6^. Polymerase-chain-reaction-based diagnostic methods provide a sensitive approach for detecting viral nucleic acids in complex biological samples but suffer from limited multiplexing capabilities^7^. There is a need for robust diagnostic methods that can simultaneously detect multiple respiratory viruses and variants in a limited sample volume, which can be quickly reconfigured to detect additional variants as they arise. Newer nucleic acid detection methods, such as nanopore sensing, which can distinguish multiple nucleic acid species^8–11^ with a unique signature for each designed DNA nanostructure may be an alternative approach for multiplexed biosensing^12–14^. Various groups have shown that nanopore sensing after viral nucleic acid enrichment or amplification may be a suitable platform to challenge these diagnostics^10,15,16^.

Here, aiming to solve many of the limitations for diagnostic multiplexing, we developed an innovative method that employs a bespoke nanobait for the simultaneous identification of multiple respiratory viruses and variants^17^. We employed programmable viral RNA cutting with ribonuclease (RNase) H to remove short RNA targets that uniquely identify the virus. The resultant RNA target is captured by the nanobait, which is immediately detected by nanopore sensing, without reverse transcription, preamplification or purification. By multiplexing several targets from the same virus in samples containing human RNA, we show that our method can increase specificity and throughput compared with existing platforms, and can pave the way for amplification-free RNA identification and diagnostics.

## Single-molecule target RNA detection with nanobaits

**Fig. 1:**
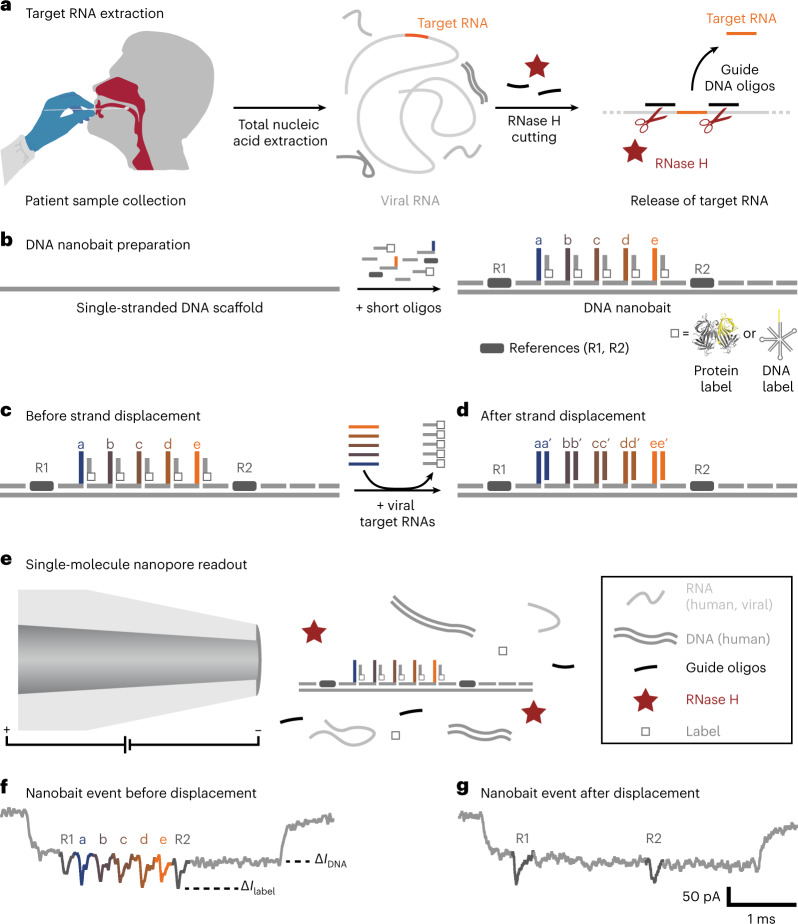
Self-assembled DNA nanobait strategy for multiplexed viral diagnostics. **a**, Oropharyngeal swab sample is collected from patients suspected to have COVID-19. The total nucleic acids were extracted (human and viral RNAs are shown in light grey; DNA is shown in dark grey), and the target RNA was cleaved out using programmable RNase H cutting. Such a treated sample was further tested for viral presence. **b**, Nanobait is made by mixing a single-stranded DNA scaffold (M13mp18 DNA) with short oligonucleotides where some of them carry complementary capture strands (a–e) for specific targets (a’–e’) in a target viral RNA. In addition, a partially complementary oligo with a structural label (protein or DNA based; white square) is added to each site to amplify the signal in nanopore recordings. We marked a sensing region with two references (dark grey). **c**,**d**, Nanobait before (**c**) and after (**d**) strand displacement reactions of five targets (coloured strands). If the targets are present, the five grey strands with labels are displaced. The two outer signals originate from reference structures that indicate the sensing region, and the five binding sites between the references are specific for the five different targets. **e**, Each nanobait is voltage driven through the nanopore and detected in a mixture of molecules. **f**, Typical nanopore current signature as a function of time for a nanobait as designed with five labels present. The first current drop corresponds to DNA (Δ*I*_DNA_) and the second to labels (Δ*I*_label_). **g**, Typical nanobait event after strand displacement of all the five targets. The presence of targets is detected by the missing downward signals specific to each target. Source data

We developed a workflow for the nanobait detection of target RNA, ranging from patient swabbing, nucleic acid extraction and programmable RNase H cutting of viral RNA (Fig. 1). The RNA targets are selected by guide oligonucleotides (single-stranded DNA, 20 nt) that were designed to bind upstream and downstream on the specific regions in a viral genome. Then, RNase H was used to digest the RNA sequence in RNA: DNA hybrids (DNA guide oligo hybridized to viral RNA segment) and release the middle target RNA (Fig. 1a, right).

The released RNA targets were identified using sequence-specific binding to the nanobait (Fig. 1b,c). The nanobait was designed (Supplementary Fig. 1) with five binding sites that could incorporate up to five targets. The nanobait was assembled by mixing a single-stranded DNA scaffold (linearized M13mp18, 7,228 nt long)^12^ with a collection of short complementary oligonucleotides (Fig. 1b, Supplementary Fig. 1 and Supplementary Table 2). Towards one end of the nanobait, the sensing region was designed to contain equally spaced sites a–e flanked by two reference structures R1 and R2, which consisted of six DNA dumbbells each (Supplementary Table 3 lists the oligonucleotides). The sensing site contained a DNA overhang, which was fully complementary to the respective target sequence. We additionally exploited a blocking oligo with a label (monovalent streptavidin^18^ or DNA flower; Supplementary Fig. 2 and Supplementary Table 1) that was only partially hybridized and left six bases unpaired. The assembly of the nanobait was confirmed by atomic force microscopy (AFM) imaging and electrophoretic mobility shift assay (Supplementary Figs. 3–5). Ultimately, if the target was present, it would bind to the six unpaired bases and displace the blocking oligo with the label at its complementary overhang, which is known as toehold-mediated strand displacement^19^. Hence, the presence of the predefined targets was indicated by the absence of a label at the respective site (Fig. 1c,d).

We determined the structure of each nanobait and ability to detect the presence or absence of targets by a single-molecule readout technique exploiting nanopore resistive pulse sensing (Fig. 1e–g). Nanopore DNA sensing works via the voltage-driven translocation of negatively charged nanobaits through a small orifice towards a positively charged electrode in an electrolyte solution (Fig. 1e)^20^. Here the nanobait translocation induces a unique current blockage signature (Fig. 1f). The first current drop corresponded with double-stranded DNA nanobait (Δ*I*_DNA_). The second current drop (Δ*I*_label_) indicated the presence of references R1 and R2 and labels a–e (Fig. 1c). Figure 1f depicts an example of a nanobait–nanopore event with seven downward spikes, where each spike corresponds to the matching colour site in the schematic shown in Fig. 1c. After strand displacement with all the five targets present (Fig. 1d, a’–e’), the five labelled oligos were displaced and only the reference spikes remain (Fig. 1g). The short duplexes were significantly smaller than the labels and not detected with these nanopores^21^. Each ionic current event on a single nanobait revealed the presence of multiple short RNA targets. The flexibility of the nanobait design permitted us to identify targets originating from multiple parts of the same virus or from multiple viral genomes.

## Simultaneous detection of multiple viral variants

**Fig. 2:**
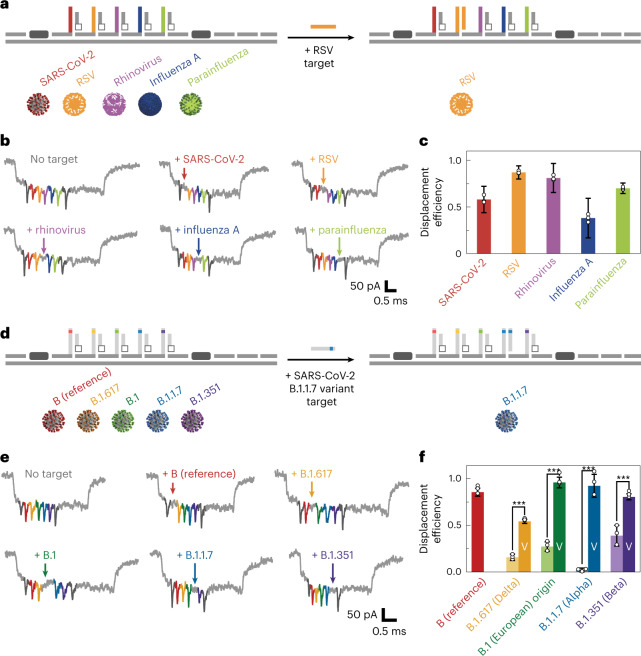
Multiplexed discrimination of viruses and SARS-CoV-2 variants with nanobait. **a**, Nanobait is designed to have five sites specific to SARS-CoV-2, RSV, rhinovirus, influenza A and parainfluenza. **b**, Example events for the condition without any targets and for each virus-specific target. The absence of the coloured spike indicates the presence of each respective target. **c**, Displacement efficiency indicates the presence of the corresponding viral target. The displacement efficiency represents a measurement with the target subtracted from the control (no targets). The error bars represent the standard error and the centre is the mean for three nanopore measurements and 50 nanopore events per measurement. **d**, Nanobait designed to detect four single-nucleotide SARS-CoV-2 variants by adaptation of the target sequences. **e**, Example events for the condition without any targets and for each variant-specific target are depicted. The absence of the coloured spike indicates the presence of each respective variant. **f,** Displacement efficiencies for single-nucleotide variants (labelled as ‘V’) are compared with the displacement efficiency for the WT strain of the SARS-CoV-2 virus isolated in Wuhan. The error bars represent the standard error and the centre is the mean for three nanopore measurements and 50 nanopore events per measurement. The difference between the conditions without and with the variant targets is statistically significant (***p < 0.001; two-sided Student’s *t*-test; *N* = 150). Source data

We designed the nanobait for the multiplexed target identification of SARS-CoV-2, respiratory syncytial virus (RSV) (universal for group A), rhinovirus (universal), influenza (universal for group A) and parainfluenza 1 (Supplementary Tables 4–7). A schematic of the nanobait design for multiple respiratory viral nucleic acid targets is shown in Fig. 2a. RSV is provided as an example of site-specific displacement (Fig. 2a). The five targets, as well as the control (no target), were independently detected using the same nanobait. The first nanopore translocation events of the nanobait in each of the individual samples are depicted in Fig. 2a and Supplementary Fig. 6. Nanopore events with seven spikes indicated the absence of targets. If the respective target for SARS-CoV-2, RSV, rhinovirus, influenza or parainfluenza were present, that spike was absent in the nanobait translocation event (Supplementary Table 4 lists the presence of targets). The displacement efficiency was calculated as the difference between a no-target control and the measurement for each site (50 nanobait events for each of the three nanopore recordings) (***p < 0.001; two-sided Student’s *t*-test) (Fig. 2c). We tested two different scenarios, with and without targets, for statistical significance.

Variant discrimination with single-nucleotide resolution is an essential feature for variant diagnostics. We tested the potential of the nanobait for the discrimination of a single-nucleotide variant by distinguishing nucleic acids from several SARS-CoV-2 variants. The five sites of nanobait allowed for the simultaneous detection of wild-type (WT) virus and four variants (Supplementary Tables 8–12 list the sequences and Supplementary Section 7 elaborate the design principles)^22^. The first site was WT SARS-CoV-2 isolated in Wuhan (B as per the PANGOLIN nomenclature)^22^. The alternative four targets were European strain B.1 and three variants of concern^17^, namely, B.1.1.7 (Alpha), B.1.351 (Beta) and B.1.617 (Delta), which were first detected in the United Kingdom, South Africa and India, respectively. As an example, we highlight the identification of the B.1.1.7 variant (Fig. 2d). We selected a variant-specific target that was fully complementary to the capture strand on the nanobait, whereas the WT target contained a mismatch in the toehold end (Supplementary Table 11). The displacement efficiency is dependent on the number and position of mismatches in the toehold domain^23^. Programming the nanobait with a single-nucleotide mismatch allowed us to discriminate the SARS-CoV-2 variant from the WT sequence. We depict example events for each sample where all the spikes are present (no targets) or the respective spike is absent depending on which variant is present (Fig. 2e; Supplementary Fig. 7 shows more events and Supplementary Table 8 lists the presence of targets). Figure 2f shows the displacement efficiency for WT targets and their corresponding variant targets for the first 50 nanobait events (coloured bars). We observed a significant difference for all the four variants compared with the respective WT samples (light- and dark-coloured bars). In addition, we demonstrated the principle by using two single-nucleotide SARS-CoV-2 RNA viral variants (Supplementary Fig. 8 and Supplementary Section 8).

## Identification of multiple SARS-CoV-2 targets

**Fig. 3:**
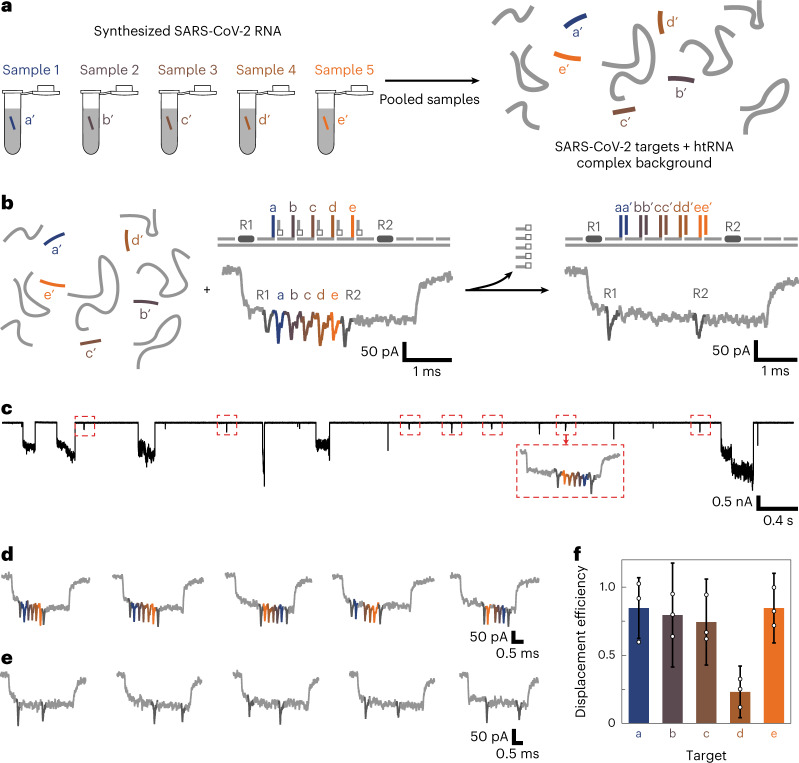
Nanobait detects multiple synthesized SARS-CoV-2 RNA targets in the background of human transcriptional RNA. **a**, Five targets from different regions in a viral genome can be separately targeted and pooled for nanopore analysis. The five targets are mixed with intact htRNA in the background to verify that viral RNA purification is not a required step. **b**, After the addition of a nanobait to the mix, all the five targets can be identified in parallel, as shown in the example events. **c**, Ionic current traces indicate the specificity of the method for the identification of nanobait-specific events even in a complex background where large downward signals originate from the background including long RNAs. All the nanobait events have been highlighted in the red dashed boxes. **d**, First five single-file nanobait events for the sample mixed with only htRNA indicate the correct current signature. **e**, First five single-file nanobait events that have been previously mixed with the targets and htRNA. All the targets are present since the corresponding spikes are absent in the nanopore events. **f**, Displacement efficiency calculated for the sample with targets added (nanobait with targets and htRNA) for all the five sites. The error bars represent the standard error and the centre is the mean for three nanopore measurements and 50 nanopore events per measurement. Source data

Diagnostic tests for viral RNA rely on multistep reactions and subsequent purification steps. We aimed to use nanobait for direct target identification without preamplification and purification. Here we used the nanobait for the specific single-molecule detection in a complex human transcriptome mixture that is human total RNA (htRNA; Invitrogen). These nanobaits could identify multiple samples from pooled samples in complex backgrounds by nanopore sensing (Fig. 3a).

We pooled together five synthesized SARS-CoV-2 RNA targets to investigate the specificity and potential crosstalk between the nanobait and non-specific htRNA background. After the targets were added, all the sites were displaced and correctly identified using the nanopore measurements (Fig. 3b). A typical current trace indicates that nanobait spikes can be easily distinguished (Fig. 3c, red boxes) from non-specific current blockages originating from the htRNA. Figure 3d,e shows the first five linear nanobait events for samples with and without targets and in the presence of htRNA; the displacement efficiency for all the five targets is depicted in Fig. 3f. Target 4 had the lowest displacement efficiency, which was in agreement with a low predicted guanine-cytosine (GC) content of 25% (ref. 24). Nanobait-based strand displacement can effectively operate even in a complex background of htRNA, oligonucleotides and proteins. We studied the kinetic details for both RNA and DNA targets and determined that 10 min was the optimal incubation time for the strand displacement reaction (Supplementary Section 9 provides the corresponding plots and events, Supplementary Table 14 lists the presence of targets and Supplementary Tables 15–17 list the target sequences and oligonucleotides).

## Design of target sites depends on viral RNA secondary structure

We next aimed to optimize multiple parameters in designing an efficient target RNA identification system. One key parameter was the successful excision of the short RNA targets from viral RNA. We found that the location of the target RNA in the viral RNA secondary structure affected the concentration of free target RNA and consequently affected the displacement efficiency. A target in a highly complementary region would remain bound to the viral RNA after cutting and prevent detection. In contrast, the release of the target after RNase H excision increases when more unpaired bases were in the target region than within the secondary structure of the viral RNA. For future experiments, we can maximize the number of unpaired bases to increase the effective concentration of the target in solution and consequently aid detection.

**Fig. 4:**
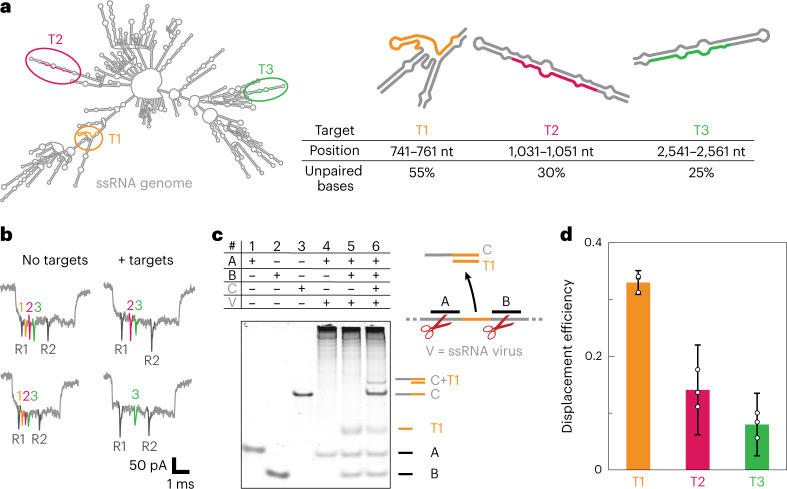
Position of a target in a viral RNA secondary structure influences the efficiency of target identification. **a**, We demonstrated that the position of a target in viral RNA correlates with the efficiency of target release after RNase H cutting. MS2 viral RNA is presented as the minimal free energy structure. Three targets (T1, T2 and T3) are selected to have different levels of paired bases for a constant 20-nt-long target. For each target, a region in the MS2 viral RNA is indicated with the percentage of unpaired bases in each target. **b**, Nanobait events with three targets present are shown, indicating the correct design (no targets). The events for the sample where the long RNA is mixed with the nanobait show displacement (+ targets). Each spike colour corresponds to a site on the nanobait. **c**, Electrophoretic mobility shift assay shows guide oligos A and B (lanes 1 and 2, respectively) and complementary oligo C to target T1 (lane 3). If only guide oligo A is used for viral RNA cutting (lane 4), there is only the oligo A band and the high-molecular-weight cut viral RNA. Once both guide oligos are added, an additional band originating from the released target T1 RNA emerges (lane 5). Once strand C is added to the same sample, we can see its shift after T1 binding (lane 6). **d**, Displacement efficiency of target RNAs correlates with the percentage of unpaired bases in a target. T1 shows the highest displacement, whereas T2 and T3 show lower displacement efficiencies. The error bars represent the standard error and the centre is the mean for three nanopore measurements and 50 nanopore events per measurement. Source data

The role of unpaired bases was demonstrated by the detection of three targets in the ~3.6 kb RNA genome of the MS2 virus (Fig. 4a shows the minimal free energy secondary structure^25^). The three targets (T1, T2 and T3) had a decreasing percentage of unpaired bases (T1, 55%; T2, 30%; and T3, 25%). Subsequently, we designed oligos and employed RNase H cutting of all the three targets and quantified the displacement efficiency using nanobait with the three sites (Fig. 4b; Supplementary Fig. 15 shows more events, Supplementary Table 18 lists the presence of targets and Supplementary Tables 19–23 list the oligonucleotides). Efficient cutting of viral RNA was confirmed by agarose gel electrophoresis (Supplementary Fig. 11). For each target, the original 3.6 kb RNA was cut into fragments of the predicted length; additionally, the predicted fragment lengths were comparable when all the three targets were simultaneously cut. We confirmed that target T1 was free in solution by hybridizing it to the complementary capture of strand C and detecting it using polyacrylamide gel electrophoresis (Fig. 4c). After cutting, target T2 was not visible by polyacrylamide gel electrophoresis (Supplementary Fig. 14), and the oligonucleotides’ running speed and non-specific interactions were validated on control polyacrylamide gel electrophoresis (Supplementary Figs. 12 and 13).

Example nanopore events and displacement efficiency with and without the targets released from the MS2 RNA genome after RNase H cutting are shown in Fig. 4b,d. The plot indicates that displacement was detected for all the three targets. Target T1 had the highest displacement efficiency, whereas target T3 had the lowest displacement efficiency. As predicted, the displacement efficiency (Fig. 4d) correlated with the unpaired base percentage in the RNA structure for each target, signifying an important design principle in selecting the viral target regions for detection.

## Amplification-free SARS-CoV-2 identification in clinical samples

**Fig. 5:**
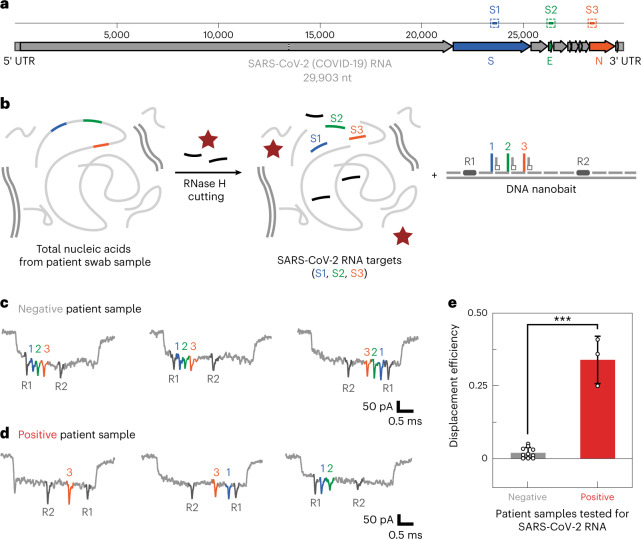
SARS-CoV-2 detection in patient’s oropharyngeal swab samples. **a**, We designed three targets (S1, S2 and S3) in conserved regions that code for spike (S), envelope (E) and nucleocapsid (N) proteins, as indicated in the schematic of the SARS-CoV-2 RNA genome (29,903 nt long). **b**, Total nucleic acids from oropharyngeal swabs contain a mix of human DNA and RNA that either have tested positive or negative for COVID-19 with qRT-PCR. The next step included RNase H target release from long RNA and mixing with the nanobait. If targets are present, they displace the oligo harbouring a label. In this way, the displacement efficiency for each site is detectable with nanopores with 1 pM of nanobait. A real-time analysis in a mixture of various biomacromolecules (human DNA, human RNA, RNase H, streptavidin and guide oligos) is directly performed without prior purification, enabling rapid nanopore readout (~10 min). **c**,**d**, Example events for both negative (**c**) and positive (**d**) SARS-CoV-2 samples. **e**, Displacement efficiency in negative samples differs from positive samples. The error bars indicate the standard error and the centre is the mean for all the events in the first 10 min. The difference between negative and positive samples has statistical significance (***p < 0.001; two-sided Student’s *t*-test). We used thirteen patient samples (*N* = 13). Source data

After establishing that RNase H had cut the MS2 RNA, we considered that the nanobait could detect SARS-CoV-2 RNA in clinical samples. We accessed oropharyngeal swabs from patients suspected to have COVID-19; the viral load of SARS-CoV-2 in oropharyngeal swabs in the clinical phase can be up to 10^8^–10^11^ copies^13,14^. The sensitivity curve for nanopore readout was plotted (Supplementary Section 16 and Supplementary Fig. 19). We used the nanobait in nucleic acid extractions from clinical samples that had been prepared for qRT-PCR (Supplementary Section 12; Supplementary Tables 24–27 list the oligos)^26^. SARS-CoV-2 targets (S1, S2 and S3) were designed in the conserved regions of the genome that contained the highest percentage of unpaired bases (Fig. 5a). S1 was in the region encoding the spike (S) protein, S2 was in the region encoding the small envelope (E) glycoprotein and S3 was in the nucleocapsid (N) protein-coding region. The total nucleic acids from the clinical samples were subjected to our RNase H protocol (Fig. 5b) and then mixed with a nanobait with sensing sites S1, S2 and S3. The reaction did not require further purification or preamplification before nanopore readout. The nanobait mixture was then analysed with nanopores containing the complex background of DNA (human and optionally DNA flower), long RNAs (human and potentially viral), short guide oligos and proteins (RNase H and monovalent streptavidin).

Nanopore events from the nanobait mixed with RNase-H-treated negative patient samples (confirmed with qRT-PCR) are shown in Fig. 5c; in addition to the two reference spikes (dark grey), three further spikes were visible and corresponded with sites for S1 (blue), S2 (green) and S3 (orange). As shown above, the nanobait current signature was not affected by the complex background or unspecific binding of DNA guide oligos. The missing spike associated with specific displacement was apparent when the nanobait was mixed with the SARS-CoV-2-positive swab samples, as confirmed by qRT-PCR (Fig. 5d). We repeated the procedure for a total of 13 SARS-CoV-2 clinical samples, which contained three positive and ten negative samples (as shown by qRT-PCR). The nanobait displacement efficiencies for negative and positive samples were consistent with the qRT-PCR results (Fig. 5e).

We additionally exploited a DNA flower as an alternative to monovalent streptavidin using patient samples processed with RNase H cutting, too. We observed comparable results with this DNA-based system (Supplementary Section 13), indicating that the detection system can be based only on DNA. An all-DNA nanobait system may aid future upscaling.

Our nanobait approach bypasses preamplification and purification and hence avoids these potentially time-consuming and expensive steps. Furthermore, the nanopore readout time can be further reduced by performing a real-time analysis on the QuipuNet convolutional neural network^27^. QuipuNet has high accuracy with an analysis speed of 1,600 events per second, which is more than sufficient for rapid viral detection. In this paper, we employed standard RNA extraction procedures for the qRT-PCR tests. The speed of the test might be further improved by using simplified RNA extraction protocols or by combining it with RNase H cutting^28,29^.

## Conclusions

Here we demonstrate the site-specific excision of a target from long viral RNA using RNase H cutting. In this way, we increase the displacement efficiency by ensuring the exact target sequence for displacement reaction in comparison to non-specific RNA fragmentation^30^. RNase H can be used to cut sequences next to a target sequence that yields new functionality besides its use in amplification-based viral detection protocols^31^. Additionally, site-specific RNA cutting can be achieved using DNAzymes or even the CRISPR/Cas system^32,33^.

Previous nanopore studies have demonstrated the ability to detect one or a limited number of short nucleic acid species in the isolated form^10,15,30,34–36^. However, the biological complexity within a test sample poses a specific challenge when wanting to discriminate targets in this complex background^11,37^. Our work demonstrates that DNA nanotechnology can be used to detect specific targets in clinical samples with nanopores. As a proof of concept, we tested the nanobait against five different respiratory viruses or SARS-CoV-2 variants in parallel. Previously, we showed that with DNA encoding, a library of 2^112^ molecules that ensures the potential to test for hundreds and thousands of viral targets in parallel can be created^12,38^, especially when multiplexed nanopore systems become more advanced.

Recent studies have developed a viral nucleic acid detection system using nanopores, which holds great promise for a rapid detection system^10,15,16^. However, preamplification and enzymatic steps in preparation for nanopore detection limit the utility of such methods, although some approaches showed the potential to omit these steps^29,30,39^. Our nanobait system does not necessitate preamplification and can identify native RNA sequences without the need for sequencing. The design of this approach overcomes an issue of non-specific spikes in nanopore measurements by using the absence of a downward spike as a positive signal for the identification of the presence of the target sequence. The nanobait demonstrated comparable features to the existing methods (Supplementary Data 1)^6,40–43^, and it can also identify multiple targets from the same viral RNA, thereby offering enhanced specificity and accuracy for viral identification, as demonstrated for the detection of SARS-CoV-2 in clinical specimens. Currently, we show that a 10 min nanobait readout with a nanopore would enable the detection of viral RNA in infectious patients with cycle threshold (CT) value of <20 (Fig. 5). Nanopores have single-molecule sensitivity; however, the number of events depends on the target concentration^35^. Hence, lower concentrations (CT > 20) can be measured by either a single nanopore running for a longer time or many nanopores in parallel. Here the detection time scales with 1/*N*, where *N* is the number of nanopores.

Rapid programmability of diagnostic platforms is of paramount importance for detecting new viruses or their variants as they arise^17^. A nanobait is rapidly adaptable for new viral targets, as we demonstrated by discriminating emerging SARS-CoV-2 variants. Our study has the potential to enable amplification-free native RNA identification. The nanobait bypasses amplification sequence biases by detecting innate RNA diversity. Our results show that a nanobait can identify short and long RNAs and may find wider applications in the analysis of physiological and pathological conditions including cancer detection.

In conclusion, we have demonstrated the simultaneous identification of nucleic acids from multiple viruses and SARS-CoV-2 RNA variants in a specific and rapid manner by combining DNA nanotechnology and nanopore sensing. We employed the easily programmable nanobait with strand displacement for discrimination between SARS-CoV-2 WT RNA from variant RNA, comprising three variants of epidemiological concern^17^. Finally, we successfully used the nanobait-based nanopore-sensing method in clinical samples and could accurately determine the presence or absence of SARS-CoV-2 in patient swabs. Nanobait circumvents the need for reverse transcription, amplification, or reaction purification, and therefore bypasses enzymatic biases and some additional steps. As the nanobait has proven to be specific and accurate for viral detection in patient samples, we think our platform can be employed for native RNA detection. The nanobait paves the way for a multiplexed amplification-free RNA detection method that is dependent only on the rapid single-molecule readout of the nanobait structure.

## Methods

### Patient sample collection

Patient samples were collected by swabbing the back of the throat (oropharyngeal swab) of patients, as previously described^26^. The samples were collected from patients with the COVID-19-like clinical picture and were tested with qRT-PCR after nucleic acid extraction. Briefly, after collection, swabs were placed into a labelled sample tube containing a lysis buffer (4 M guanidine thiocyanate, 25 mM Tris–HCl, 0.5% β-mercaptoethanol and MS2 RNA (200 ng µl^–1^; Roche)). The tube was gently agitated to ensure the even distribution of lysis buffer. The safety steps have been previously described and were performed in a certified CL2 laboratory^26^.

### Nucleic acid extraction

The total nucleic acid was extracted using spin-column-based systems and as employed by standardized qRT-PCR testing^26^. The internal amplification control (MS2 (~6 × 10^4^ PFU ml^–1^) per 10 ml of lysis buffer) was added in the top-up lysis buffer (25 µl per 10 ml of lysis buffer). The sample was eluted in 100 µl of nuclease-free water (nfH_2_O; Invitrogen) and left to stand for 1 min before centrifugation for 1 min at 21,130×*g* (15,000 rpm) in a benchtop microfuge. The eluted samples were directly subjected to qRT-PCR. The remaining nucleic acid extracts were stored at −80 °C and further used for nanobait–nanopore sensing.

### qRT-PCR for SARS-CoV-2

SARS-CoV-2 detection was performed as previously described^26^. Per reaction, the master mix contained 12.5 µl of 2× Luna Universal Probe One-Step reaction mix, 0.5 µl of 20 µM Wu forward primer (5′-ATGGGTTGGGATTATCCTAAATGTGA-3′), 0.5 µl of 20 µM Wu reverse primer (5′-GCAGTTGTGGCATCTCCTGATGAG-3′), 0.3 µl of 10 µM MGB Probe 3 fluorescein (5′-ATGCTTAGAATTATGGCCTCAC-3′), 0.5 µl of 10 µM of internal control forward primer for MS2 RNA, 0.5 µl of 10 µM internal control reverse primer for MS2 RNA, 0.3 µl of 10 µM internal probe (MS2 ROX), 1 µl of Luna WarmStart RT Enzyme Mix and 3.9 µl of nfH_2_O. Then, 20 µl of the master mix was aliquoted into each well of a 96-well plate and then combined with 5 µl of each extract. The MS2 internal extraction and amplification control that underwent the full extraction protocol was included as the negative extraction control in a minimum of two wells on the qRT-PCR plate. To determine potential contamination in the qRT-PCR process, 5 µl nfH_2_O was included as the qRT-PCR negative control. Then, 5 µl of spiked SARS-CoV-2 template plasmid was included in a single well as the qRT-PCR positive control. After adding 5 µl of each sample to its designated well, the plate was sealed with an optically clear plastic seal. The plate was centrifuged for 1 min at 2,000×*g* (1,000 rpm) at 4 °C and then inserted in the qRT-PCR machine (QuantStudio, Thermo Fisher Scientific) and the run was parametrized. Signals for fluorescein (FAM) and carboxyrhodamine (ROX) were acquired. ROX was used to detect the internal MS2 control and fluorescein was used to detect SARS-CoV-2 RNA. The assay was performed for 2 min at 25 °C, 15 min at 50 °C (for the reverse transcriptase), 2 min at 90 °C, before 45 cycles of 95 °C for 3 s followed by 60 °C for 30 s. The results were determined by the confirmation of correct positive controls (amplification of the plasmid), extraction and amplification controls of all the samples (ROX channel), no amplification in the negative controls and consistent mean values of controls. SARS-CoV-2 positivity was confirmed by amplification in the fluorescein channel with an appropriate sigmoidal curve with a CT value of ≤36. The CT values of MS2 and MGB probe 3 were maintained to track the quality and reproducibility of the assay^44^.

### Programmable RNase H cutting for nanobait

For nanopore sensing, SARS-CoV-2 RNA controls, nucleic acid extracts (patient samples) or MS2 viral RNA were used further for detection with nanobait. First, we mixed guide oligos with the sample and heated it to 70 °C for 5 min. RNase H (5,000 units per ml; NEB) was added, mixed and heated for 20 min at 37 °C to allow the enzyme to cut RNA in the DNA: RNA hybrid that effectively releases the target RNA. RNase H was thermally inactivated by incubation at 65 °C for 10 min. Guide oligos were validated to not form intramolecular structures, homo- or heterodimers using the NUPACK software^45^. For the measurement with the absent target, the same protocol including guide oligos was used. The control measurements show no displacement, and hence, we can exclude any substantial cross-binding from guide oligos.

### Viral target sequence properties for nanobait

The length of target, toehold length and GC content were selected to ensure optimal hybridization^21^. For the DNA nanobait designs, the target sequences were selected to be in the conserved regions of a viral genome and had 40–60% GC content to form a stable 20 bp duplex. The toehold length was selected to be 6 nt long and have 40–60% GC content. We tested all the sequences for potential undesirable highly stable intramolecular interactions or homodimers using the NUPACK software (web application 2020)^45^. Then, we performed a cross-reactivity check between multiple sites employed in each experiment^45^.

### Preparation of DNA flower for nanobait

We designed a DNA flower as another label for SARS-CoV-2 RNA detection from the patient samples. Three DNA flowers specific for each SARS-CoV-2 target (seven-way junctions, 7WJa, 7WJb and 7WJc) were separately prepared. Taking 7WJc as an example, 4 μM DNA strand J1, J2, J3 and J4c (Supplementary Table 1) were mixed together in TM buffer (10 mM Tris–HCl, 10 mM MgCl_2_, pH 8.0) and heated to 90 °C for 5 min, then cooled down to 65 °C for 15 min, 45 °C for 15 min, 37 °C for 20 min, 25 °C for 20 min and finally to 4 °C for 20 min. Strand J4c was substituted by J4b to prepare 7WJb. For 7WJa, to avoid self-folding at site 43 on the nanobait, J1, J2, J3 J4a and C43 were mixed together before annealing.

### Self-assembly of DNA nanobait

The DNA nanobait was assembled by mixing linearized single-stranded M13 DNA (M13mp18, 7,249 nt, Guild Biosciences, 100 nM) with short complementary oligonucleotides^12^ (some of which harboured reference structures and capture strands) and by adding partially complementary strands that were 3′-biotinylated for toehold-mediated strand displacement reaction. The linearized M13 DNA (7,228 nt in length) was complemented by oligonucleotides, thereby creating a nicked double-stranded nanobait with two-terminal four deoxythymidine overhangs that prevent multimerization^12^. The mix contained 20 nM of linearized M13 DNA, 60 nM of oligonucleotides (three times excess to M13 DNA), 3′-biotinylated strands in the concentration of 180 nM, 10 mM MgCl_2_ and 1× TE (10 mM Tris–HCl, 1 mM EDTA, pH 8.0). It was mixed by pipetting and spun down before heating to 70 °C for 30 s and cooled down over 45 min to ambient temperature. Excess oligonucleotides were removed using Amicon Ultra 0.5 ml centrifugal filters with 100 kDa cutoff with a washing buffer (10.0 mM Tris–HCl pH 8.0, 0.5 mM MgCl_2_). If DNA flowers were employed as a label, the partially complementary strands that carry it were incubated in 10 mM MgCl_2_ for 2 h at ambient temperature, and subsequently, Amicon filtration was performed as described above. The asymmetry of the nanobait design allows for the unambiguous identification of the binding sites. The nanobait was stored until used for further experiments under 4–10 °C in 0.5 mM MgCl_2_, 10.0 mM Tris–HCl, pH 8.0. The nanobait design was checked by nanopore readout before each measurement.

### Nanopore readout of DNA nanobait

The nanobait was mixed with a sample (nucleic acid extract or purified viral targets at ten times excess) in 10 mM MgCl_2_ and 100 mM NaCl. The mixture (5 μl) was incubated at room temperature (~10 min) until prepared for nanopore measurement. The difference in the target sequence composition and its physical characteristics might lead to variability in hybridization and hence the displacement efficiency of sensing sites^21^. We have used htRNA (100 ng μl^–1^; Invitrogen) as a background where indicated, to show that there are no non-specific signals induced by human native RNAs. For nanopore measurement, the sample was diluted to <0.5 nM nanobait (for purified viral targets) or 4.7 μl of RNase-H-cut patient sample was mixed with 0.3 μl of monovalent streptavidin (SAe1D3)^18^ (1 μM), 5 μl of LiCl (4.0 M) and 5.0 μl of LiCl (8.0 M). We have fabricated 14 ± 3 nm (mean ± standard deviation) nanopores^12^ using quartz glass capillaries with 0.5 mm outer diameter and 0.2 mm inner diameter (Sutter Instrument) by laser-assisted puller P-2000 (Sutter Instrument). The mix was pipetted in a nanopore polydimethylsiloxane chip, and all the measurements were performed at a constant voltage of 600 mV. Nanopore measurement details are shown in Supplementary Table 30.

### Real-time nanopore data analysis

Nanopore data analysis is explained in detail in Supplementary Section 14. Briefly, nanobait events were filtered out of raw ionic current traces and then the detection region was determined, and information of the spike’s presence at each specific site was extracted. The plotted displacement efficiency was calculated as a displacement efficiency for a measurement subtracted to a no-target control for each site (50 nanobait events for each of the three nanopore recordings), unless stated otherwise:

$$\begin{array}{l}{\mathrm{Displacement}}\,{\mathrm{efficiency}} =\frac{1}{3}\mathop {\sum}\limits_{n = 1}^3 \left\{ {1 -\frac{1}{{50}}\mathop {\sum}\limits_{n = 1}^{50} {\left[ {f\left( n \right) = \left( {\frac{{1,\,\mathrm{peak}}}{{0,\,{\mathrm{no}}\,{\mathrm{peak}}}}} \right)} \right]_{{{{\mathrm{target}}}}}} } \right\}\\ - \frac{1}{3}\mathop {\sum}\limits_{n = 1}^3 {\left\{ {1 - \frac{1}{{50}}\mathop {\sum }\limits_{n = 1}^{50} \left[ {f\left( n \right) = \left( {\frac{{1,\,\mathrm{peak}}}{{0,\,\mathrm{no}}\,{\mathrm{peak}}}} \right)} \right]_{{{{\mathrm{no}}}}\,{{{\mathrm{target}}}}}} \right\}} \end{array}.$$

We verified that the convolutional neural network QuipuNet^27^ was capable of the real-time analysis of nanopore data following the described procedure. Previously, we demonstrated that with around ten events, we reach 99% confidence in a positive detection of our designed DNA structures^46^.

### AFM imaging

AFM (Nanosurf Mobile S) imaging of nanobaits was performed in air in the non-contact mode. The nanobait structures were diluted to 1 ng μl^–1^ in 1 mM MgCl_2_ and 10 μl was added to freshly cleaved mica, incubated for 1 min, rinsed with filtered Milli-Q water and then blow dried with nitrogen. Before scanning, the mica plate was affixed to the AFM sample stage using double-sided adhesive tape. Image visualization and analysis were performed using Gwyddion (version 2.60).

### Statistical analysis

For all the measurements, 99.9% confidence intervals for displacement efficiencies were calculated. Statistical significance between two sites without and with the target was tested using a two-sided Student’s *t*-test.

### Reporting summary

Further information on research design is available in the Nature Portfolio Reporting Summary linked to this article.

## Online content

Any methods, additional references, Nature Portfolio reporting summaries, source data, extended data, supplementary information, acknowledgements, peer review information; details of author contributions and competing interests; and statements of data and code availability are available at 10.1038/s41565-022-01287-x.

## Supplementary information

Supplementary Information Supplementary Sections 1–16, Figs. 1–19, Tables 1–30 and refs. 1–20.

Reporting Summary

Supplementary Data Methods compared with nanobait.

## Data Availability

Data supporting the findings of this study are available in the main text and Supplementary Information. Additional raw data are available at 10.17863/CAM.89753. Source data are provided with this paper.

## Source data

Source Data Fig. 1 Statistical source data and raw ionic current data.
Source Data Fig. 2 Statistical source data and raw ionic current data.
Source Data Fig. 3 Statistical source data and raw ionic current data.
Source Data Fig. 4 Statistical source data, raw ionic current data, and predicted structure and sequence used.
Source Data Fig. 5 Statistical source data and raw ionic current data.
