# Supplementary material for: Simultaneous identification of viruses and viral variants with programmable DNA nanobait
Source: Nat Nanotechnol. 2023 Jan 16;18(3):290–8. doi: 10.1038/s41565-022-01287-x (PMC10020084; doi:10.1038/s41565-022-01287-x)
Supplement: Supplementary file 2 — Reporting Summary [file 41565_2022_1287_MOESM2_ESM.pdf]

nature portfolio

Corresponding author(s): Ulrich F. Keyser

Last updated by author(s): Oct 20, 2022

Reporting Summary

Nature Portfolio wishes to improve the reproducibility of the work that we publish. This form provides structure for consistency and transparency in reporting. For further information on Nature Portfolio policies, see our Editorial Policies and the Editorial Policy Checklist.

Statistics

For all statistical analyses, confirm that the following items are present in the figure legend, table legend, main text, or Methods section.

n/a	Confirmed	
☐	☒	The exact sample size (n) for each experimental group/condition, given as a discrete number and unit of measurement
☐	☒	A statement on whether measurements were taken from distinct samples or whether the same sample was measured repeatedly
☐	☒	The statistical test(s) used AND whether they are one- or two-sided<br>*Only common tests should be described solely by name; describe more complex techniques in the Methods section.*
☒	☐	A description of all covariates tested
☒	☐	A description of any assumptions or corrections, such as tests of normality and adjustment for multiple comparisons
☐	☒	A full description of the statistical parameters including central tendency (e.g. means) or other basic estimates (e.g. regression coefficient) AND variation (e.g. standard deviation) or associated estimates of uncertainty (e.g. confidence intervals)
☐	☒	For null hypothesis testing, the test statistic (e.g. F, t, r) with confidence intervals, effect sizes, degrees of freedom and P value noted<br>*Give P values as exact values whenever suitable.*
☒	☐	For Bayesian analysis, information on the choice of priors and Markov chain Monte Carlo settings
☒	☐	For hierarchical and complex designs, identification of the appropriate level for tests and full reporting of outcomes
☒	☐	Estimates of effect sizes (e.g. Cohen's d, Pearson's r), indicating how they were calculated

Our web collection on statistics for biologists contains articles on many of the points above.

Software and code

Policy information about availability of computer code

Data collection	The data was collected using software written with LabView 2013 (National Instruments). Nupack online (http://www.nupack.org/).
Data analysis	Data analysis was also performed using software written with LabView 2013 (National Instruments).

For manuscripts utilizing custom algorithms or software that are central to the research but not yet described in published literature, software must be made available to editors and reviewers. We strongly encourage code deposition in a community repository (e.g. GitHub). See the Nature Portfolio guidelines for submitting code & software for further information.

Data

Policy information about availability of data

All manuscripts must include a data availability statement. This statement should provide the following information, where applicable:

- Accession codes, unique identifiers, or web links for publicly available datasets
- A description of any restrictions on data availability
- For clinical datasets or third party data, please ensure that the statement adheres to our policy

Raw data of ionic current values for translocations together with a table summarizing all the nanopores used is available at the supplementary information and source data are available online via the repository. SARS-CoV-2 detection in clinical samples and tested with RT-qPCR are available here https://wellcomeopenresearch.org/articles/5-110/v2. Our data can be analysed with any software that allows for peak detection and even more importantly by hand.

Field-specific reporting

Please select the one below that is the best fit for your research. If you are not sure, read the appropriate sections before making your selection.

☒ Life sciences ☐ Behavioural & social sciences ☐ Ecological, evolutionary & environmental sciences

For a reference copy of the document with all sections, see nature.com/documents/nr-reporting-summary-flat.pdf

Life sciences study design

All studies must disclose on these points even when the disclosure is negative.

Sample size	We have measured on each nanopore device as long as the device are usable. Briefly, nanobait events were filtered out of raw ionic current traces, then the detection region was determined, and information of the spike's presence at each specific site was extracted. The displacement efficiency was calculated as a difference between a no target control and a measurement for each site. We measured during the lifetime of nanopore and first fifty nanobait events for each of three nanopore recordings were further used for analysis. 99.9% confidence intervals for the displacement efficiencies were calculated for all measurements. The statistical significance between two sites without and with the target was tested using a student's T-test.
Data exclusions	Our data analysis exclude fragments of DNA, cut RNA, proteins as well as aggregates that are excluded based on event charge deficit of nanopore event and the absence of reference structures.
Replication	The measurements presented in the main text were repeated using at least three different nanopores and experiments. These results are presented in the supplementary information. Each repeat successfully confirmed the central findings.
Randomization	Randomization was not relevant to this study since we were not comparing experimental groups rather demonstrating method applicability.
Blinding	Blinding was not relevant to this study since we were not comparing experimental groups. We did blind study for the patient samples analysis.

Reporting for specific materials, systems and methods

We require information from authors about some types of materials, experimental systems and methods used in many studies. Here, indicate whether each material, system or method listed is relevant to your study. If you are not sure if a list item applies to your research, read the appropriate section before selecting a response.

Materials & experimental systems

n/a	Involved in the study	
☒	☐	Antibodies
☒	☐	Eukaryotic cell lines
☒	☐	Palaeontology and archaeology
☒	☐	Animals and other organisms
☒	☐	Human research participants
☒	☐	Clinical data
☒	☐	Dual use research of concern

Methods

n/a	Involved in the study	
☒	☐	ChIP-seq
☒	☐	Flow cytometry
☒	☐	MRI-based neuroimaging
