# Supplementary material for: Simultaneous identification of viruses and viral variants with programmable DNA nanobait
Source: Nat Nanotechnol. 2023 Jan 16;18(3):290–8. doi: 10.1038/s41565-022-01287-x (PMC10020084; doi:10.1038/s41565-022-01287-x)
Supplement: Source Data Fig. 4 — Statistical source data, raw ionic current data, and predicted structure and sequence used. [file 41565_2022_1287_MOESM7_ESM.zip › Figure 4 - Copy/Figure 4a/Figure 4_MS2 RNA MFE prediction details.docx]

Phage MS2 nucleotide sequence, NC_001417.2. was used to predict secondary structure using RNAfold WebServer, RNAfold version 2.4.18 (http://rna.tbi.univie.ac.at//cgi-bin/RNAWebSuite/RNAfold.cgi; Date of access the 13^th^ of May 2021).

In the folder there are the following files:

- Unaltered MFE plain structure drawing (file name “**MS2 RNA MFE_ss.eps**”)
- Unaltered MFE structure drawing encoding base-pair probabilities (file name “**MS2 RNA MFE_pp.eps**”)
- Unaltered MFE structure drawing encoding positional entropy (file name “**MS2 RNA MFE_pe.eps**”)
- A mountain plot representation of the MFE structure, the thermodynamic ensemble of RNA structures, and the centroid structure as well as the positional entropy for each position (file name “**MS2 RNA MFE_mountain.eps**”)

RNA parameters are described in:

Mathews DH, Disney MD, Childs JL, Schroeder SJ, Zuker M, Turner DH. (2004) Incorporating chemical modification constraints into a dynamic programming algorithm for prediction of RNA secondary structure. *Proc Natl Acad Sci U S A* 101(19):7287-92.

References:

1. Gruber AR, Lorenz R, Bernhart SH, Neuböck R, Hofacker IL.The Vienna RNA Websuite. *Nucleic Acids Research*, Volume 36, Issue suppl_2, 1 July 2008, Pages W70-W74, DOI: 10.1093/nar/gkn188
2. Lorenz, R. and Bernhart, S.H. and Höner zu Siederdissen, C. and Tafer, H. and Flamm, C. and Stadler, P.F. and Hofacker, I.L. "ViennaRNA Package 2.0", *Algorithms for Molecular Biology*, 6:1 page(s): 26, 2011
