# Supplementary material for: Simultaneous identification of viruses and viral variants with programmable DNA nanobait
Source: Nat Nanotechnol. 2023 Jan 16;18(3):290–8. doi: 10.1038/s41565-022-01287-x (PMC10020084; doi:10.1038/s41565-022-01287-x)
Supplement: Source Data Fig. 4 — Statistical source data, raw ionic current data, and predicted structure and sequence used. [file 41565_2022_1287_MOESM7_ESM.zip › Figure 4 - Copy/Figure 4a/Figure 4_MS2 RNA nucleotide sequence.docx]

Phage MS2 nucleotide sequence, NC_001417.2

GGGTGGGACCCCTTTCGGGGTCCTGCTCAACTTCCTGTCGAGCTAATGCCATTTTTAATGTCTTTAGCGAGACGCTACCATGGCTATCGCTGTAGGTAGCCGGAATTCCATTCCTAGGAGGTTTGACCTGTGCGAGCTTTTAGTACCCTTGATAGGGAGAACGAGACCTTCGTCCCCTCCGTTCGCGTTTACGCGGACGGTGAGACTGAAGATAACTCATTCTCTTTAAAATATCGTTCGAACTGGACTCCCGGTCGTTTTAACTCGACTGGGGCCAAAACGAAACAGTGGCACTACCCCTCTCCGTATTCACGGGGGGCGTTAAGTGTCACATCGATAGATCAAGGTGCCTACAAGCGAAGTGGGTCATCGTGGGGTCGCCCGTACGAGGAGAAAGCCGGTTTCGGCTTCTCCCTCGACGCACGCTCCTGCTACAGCCTCTTCCCTGTAAGCCAAAACTTGACTTACATCGAAGTGCCGCAGAACGTTGCGAACCGGGCGTCGACCGAAGTCCTGCAAAAGGTCACCCAGGGTAATTTTAACCTTGGTGTTGCTTTAGCAGAGGCCAGGTCGACAGCCTCACAACTCGCGACGCAAACCATTGCGCTCGTGAAGGCGTACACTGCCGCTCGTCGCGGTAATTGGCGCCAGGCGCTCCGCTACCTTGCCCTAAACGAAGATCGAAAGTTTCGATCAAAACACGTGGCCGGCAGGTGGTTGGAGTTGCAGTTCGGTTGGTTACCACTAATGAGTGATATCCAGGGTGCATATGAGATGCTTACGAAGGTTCACCTTCAAGAGTTTCTTCCTATGAGAGCCGTACGTCAGGTCGGTACTAACATCAAGTTAGATGGCCGTCTGTCGTATCCAGCTGCAAACTTCCAGACAACGTGCAACATATCGCGACGTATCGTGATATGGTTTTACATAAACGATGCACGTTTGGCATGGTTGTCGTCTCTAGGTATCTTGAACCCACTAGGTATAGTGTGGGAAAAGGTGCCTTTCTCATTCGTTGTCGACTGGCTCCTACCTGTAGGTAACATGCTCGAGGGCCTTACGGCCCCCGTGGGATGCTCCTACATGTCAGGAACAGTTACTGACGTAATAACGGGTGAGTCCATCATAAGCGTTGACGCTCCCTACGGGTGGACTGTGGAGAGACAGGGCACTGCTAAGGCCCAAATCTCAGCCATGCATCGAGGGGTACAATCCGTATGGCCAACAACTGGCGCGTACGTAAAGTCTCCTTTCTCGATGGTCCATACCTTAGATGCGTTAGCATTAATCAGGCAACGGCTCTCTAGATAGAGCCCTCAACCGGAGTTTGAAGCATGGCTTCTAACTTTACTCAGTTCGTTCTCGTCGACAATGGCGGAACTGGCGACGTGACTGTCGCCCCAAGCAACTTCGCTAACGGGGTCGCTGAATGGATCAGCTCTAACTCGCGTTCACAGGCTTACAAAGTAACCTGTAGCGTTCGTCAGAGCTCTGCGCAGAATCGCAAATACACCATCAAAGTCGAGGTGCCTAAAGTGGCAACCCAGACTGTTGGTGGTGTAGAGCTTCCTGTAGCCGCATGGCGTTCGTACTTAAATATGGAACTAACCATTCCAATTTTCGCTACGAATTCCGACTGCGAGCTTATTGTTAAGGCAATGCAAGGTCTCCTAAAAGATGGAAACCCGATTCCCTCAGCAATCGCAGCAAACTCCGGCATCTACTAATAGACGCCGGCCATTCAAACATGAGGATTACCCATGTCGAAGACAACAAAGAAGTTCAACTCTTTATGTATTGATCTTCCTCGCGATCTTTCTCTCGAAATTTACCAATCAATTGCTTCTGTCGCTACTGGAAGCGGTGATCCGCACAGTGACGACTTTACAGCAATTGCTTACTTAAGGGACGAATTGCTCACAAAGCATCCGACCTTAGGTTCTGGTAATGACGAGGCGACCCGTCGTACCTTAGCTATCGCTAAGCTACGGGAGGCGAATGGTGATCGCGGTCAGATAAATAGAGAAGGTTTCTTACATGACAAATCCTTGTCATGGGATCCGGATGTTTTACAAACCAGCATCCGTAGCCTTATTGGCAACCTCCTCTCTGGCTACCGATCGTCGTTGTTTGGGCAATGCACGTTCTCCAACGGTGCTCCTATGGGGCACAAGTTGCAGGATGCAGCGCCTTACAAGAAGTTCGCTGAACAAGCAACCGTTACCCCCCGCGCTCTGAGAGCGGCTCTATTGGTCCGAGACCAATGTGCGCCGTGGATCAGACACGCGGTCCGCTATAACGAGTCATATGAATTTAGGCTCGTTGTAGGGAACGGAGTGTTTACAGTTCCGAAGAATAATAAAATAGATCGGGCTGCCTGTAAGGAGCCTGATATGAATATGTACCTCCAGAAAGGGGTCGGTGCTTTCATCAGACGCCGGCTCAAATCCGTTGGTATAGACCTGAATGATCAATCGATCAACCAGCGTCTGGCTCAGCAGGGCAGCGTAGATGGTTCGCTTGCGACGATAGACTTATCGTCTGCATCCGATTCCATCTCCGATCGCCTGGTGTGGAGTTTTCTCCCACCAGAGCTATATTCATATCTCGATCGTATCCGCTCACACTACGGAATCGTAGATGGCGAGACGATACGATGGGAACTATTTTCCACAATGGGAAATGGGTTCACATTTGAGCTAGAGTCCATGATATTCTGGGCAATAGTCAAAGCGACCCAAATCCATTTTGGTAACGCCGGAACCATAGGCATCTACGGGGACGATATTATATGTCCCAGTGAGATTGCACCCCGTGTGCTAGAGGCACTTGCCTACTACGGTTTTAAACCGAATCTTCGTAAAACGTTCGTGTCCGGGCTCTTTCGCGAGAGCTGCGGCGCGCACTTTTACCGTGGTGTCGATGTCAAACCGTTTTACATCAAGAAACCTGTTGACAATCTCTTCGCCCTGATGCTGATATTAAATCGGCTACGGGGTTGGGGAGTTGTCGGAGGTATGTCAGATCCACGCCTCTATAAGGTGTGGGTACGGCTCTCCTCCCAGGTGCCTTCGATGTTCTTCGGTGGGACGGACCTCGCTGCCGACTACTACGTAGTCAGCCCGCCTACGGCAGTCTCGGTATACACCAAGACTCCGTACGGGCGGCTGCTCGCGGATACCCGTACCTCGGGTTTCCGTCTTGCTCGTATCGCTCGAGAACGCAAGTTCTTCAGCGAAAAGCACGACAGTGGTCGCTACATAGCGTGGTTCCATACTGGAGGTGAAATCACCGACAGCATGAAGTCCGCCGGCGTGCGCGTTATACGCACTTCGGAGTGGCTAACGCCGGTTCCCACATTCCCTCAGGAGTGTGGGCCAGCGAGCTCTCCTCGGTAGCTGACCGAGGGACCCCCGTAAACGGGGTGGGTGTGCTCGAAAGAGCACGGGTGCGAAAGCGGTCCGGCTCCACCGAAAGGTGGGCGGGCTTCGGCCCAGGGACCTCCCCCTAAAGAGAGGACCCGGGATTCTCCCGATTTGGTAACTAGCTGCTTGGCTAGTTACCACCCA
